# RETRACTION: A Meta‐Analysis Examined the Effect of Intrawound Vancomycin on Surgical Site Wound Infections in Non‐Spinal Neurosurgical Operation

**DOI:** 10.1111/iwj.70507

**Published:** 2025-04-21

## Abstract

**RETRACTION:** ZengX. , YangB. , ZhangB. , XuB. , RongC. , SheJ. , GuoW. , KongJ. , LiuY. , ZhaoD. , and XuX. , “A Meta‐Analysis Examined the Effect of Intrawound Vancomycin on Surgical Site Wound Infections in Non‐Spinal Neurosurgical Operation,” International Wound Journal 20, no. 5 (2023): 1584–1590, 10.1111/iwj.14014.36424840 PMC10088818

The above article, published online on 24 November 2022, in Wiley Online Library (http://onlinelibrary.wiley.com/), has been retracted by agreement between the journal Editor in Chief, Professor Keith Harding; and John Wiley & Sons Ltd. Following an investigation by the publisher, all parties have concluded that this article was accepted solely on the basis of a compromised peer review process. In addition, the investigation found significant textual overlap between this article and another article by different authors [1]. The editors have therefore decided to retract the article. The authors did not respond to our notice regarding the retraction.

Reference

[1] BokhariR. , YouE. , ZeilerF. A. , BakhaidarM. , BajunaidK. , LasryO. , BaeesaS. , and MarcouxJ. , “Effect of Intrawound Vancomycin on Surgical Site Infections in Nonspinal Neurosurgical Procedures: A Systematic Review and Meta‐Analysis,” World Neurosurgery 97 (2019): 409–417, 10.1016/j.wneu.2018.10.168.30391768

**RETRACTION:** X. Zeng , B. Yang , B. Zhang , B. Xu , C. Rong , J. She , W. Guo , J. Kong , Y. Liu , D. Zhao , and X. Xu , “A Meta‐Analysis Examined the Effect of Intrawound Vancomycin on Surgical Site Wound Infections in Non‐Spinal Neurosurgical Operation,” International Wound Journal 20, no. 5 (2023): 1584–1590, 10.1111/iwj.14014.36424840 PMC10088818

The above article, published online on 24 November 2022, in Wiley Online Library (http://onlinelibrary.wiley.com/), has been retracted by agreement between the journal Editor in Chief, Professor Keith Harding; and John Wiley & Sons Ltd. Following an investigation by the publisher, all parties have concluded that this article was accepted solely on the basis of a compromised peer review process. In addition, the investigation found significant textual overlap between this article and another article by different authors [1]. The editors have therefore decided to retract the article. The authors did not respond to our notice regarding the retraction.

**Reference**

[1] R. Bokhari , E. You , F. A. Zeiler , M. Bakhaidar , K. Bajunaid , O. Lasry , S. Baeesa , and J. Marcoux , “Effect of Intrawound Vancomycin on Surgical Site Infections in Nonspinal Neurosurgical Procedures: A Systematic Review and Meta‐Analysis,” World Neurosurgery 97 (2019): 409–417, 10.1016/j.wneu.2018.10.168.30391768

